# The mir-423-5p/MMP-2 Axis Regulates the Nerve Growth Factor-Induced Promotion of Chondrosarcoma Metastasis

**DOI:** 10.3390/cancers13133347

**Published:** 2021-07-03

**Authors:** Huey-En Tzeng, Syuan-Ling Lin, Louis-Anoop Thadevoos, Chih-Yuan Ko, Ju-Fang Liu, Yu-Wen Huang, Chih-Yang Lin, Yi-Chin Fong, Chih-Hsin Tang

**Affiliations:** 1 Program for Cancer Molecular Biology and Drug Discovery, College of Medical Science and Technology, Taipei Medical University, Taipei 11031, Taiwan; tzhuen@tmu.edu.tw; 2 Graduate Institute of Cancer Biology and Drug Discovery, College of Medical Science and Technology, Taipei Medical University, Taipei 11031, Taiwan; 3 Division of Hematology/Oncology, Department of Medicine, Taipei Medical University Hospital, Taipei 11031, Taiwan; 4 School of Medicine, College of Medicine, Taipei Medical University, Taipei 11031, Taiwan; 5 Translational Medicine Research Center, China Medical University Hospital, Taichung 40402, Taiwan; T34946@mail.cmuh.org.tw; 6 International Master Program of Biomedical Sciences, China Medical University, Taichung 40402, Taiwan; u107206102@cmu.edu.tw; 7 Department of Orthopedic Surgery, China Medical University Hospital, Taichung 40402, Taiwan; D14333@mail.cmuh.org.tw; 8 School of Oral Hygiene, College of Oral Medicine, Taipei Medical University, Taipei 11031, Taiwan; Jufangliu@tmu.edu.tw; 9 Graduate Institute of Biomedical Sciences, China Medical University, Taichung 40402, Taiwan; u105305004@cmu.edu.tw; 10 Department of Pharmacology, School of Medicine, China Medical University, Taichung 40402, Taiwan; u9957651@cmu.edu.tw; 11 Department of Sports Medicine, College of Health Care, China Medical University, Taichung 40402, Taiwan; 12 Department of Orthopedic Surgery, China Medical University Beigang Hospital, Yunlin 65152, Taiwan; 13 Department of Biotechnology, College of Health Science, Asia University, Taichung 41354, Taiwan; 14 Chinese Medicine Research Center, China Medical University, Taichung 40402, Taiwan

**Keywords:** NGF, chondrosarcoma, metastasis, MMP-2, miR-423-5p

## Abstract

**Simple Summary:**

A chondrosarcoma is a common tumor of the bone that has a high propensity to metastasize to distant organs. The effects of NGF in a chondrosarcoma are not confirmed although NGF is capable of promoting the progression and metastasis of several different types of tumors. Here, we found that NGF promotes the chondrosarcoma migration and metastasis in vitro and in vivo. The levels of NGF and MMP-2 in human chondrosarcoma tumor tissues correlated strongly with the tumor stage. We identified that NGF induces the MMP-2 synthesis and chondrosarcoma cell motility by inhibiting miR-423-5p expression through the FAK and c-Src pathways. We suggest that NGF is a worthwhile therapeutic target in the treatment of a metastatic chondrosarcoma.

**Abstract:**

A chondrosarcoma is a common tumor of the soft tissue and bone that has a high propensity to metastasize to distant organs. Nerve growth factor (NGF) is capable of promoting the progression and metastasis of several different types of tumors although the effects of NGF in a chondrosarcoma are not confirmed. Here, we found that the levels of NGF and matrix metalloproteinase-2 (MMP-2) correlated with the tumor stage in patients with a chondrosarcoma. NGF facilitated the MMP-2-dependent cellular migration in human chondrosarcoma JJ012 cells while the overexpression of NGF enhanced the lung metastasis in a mouse model of a chondrosarcoma. NGF promoted the MMP-2 synthesis and cell migration by inhibiting miR-423-5p expression through the FAK and c-Src signaling cascades. NGF appears to be a worthwhile therapeutic target in the treatment of a metastatic chondrosarcoma.

## 1. Introduction

A chondrosarcoma is a common tumor of the soft tissue and bone that occurs typically in cartilage-enriched bone (e.g., the femur, tibia or pelvis) [1,2] and has a high propensity to metastasize to distant organs [1]. High grade chondrosarcomas are particularly prone to metastasize to the lungs [3,4] so therapeutic strategies that delay or inhibit this phenomenon will improve patient survival. The metastatic process involves the secretion of proteolytic enzymes such as matrix metalloproteinases (MMPs) and cathepsins, capable of degrading the extracellular matrix (ECM) and basement membrane [5,6]. It is likely that the ECM component alpha-1 type I collagen (col1a1) contributes to the tumor growth as col1a1 is upregulated in various tumors [7] and significantly higher levels of MMP-2 expression have not only been recorded in human chondrosarcoma specimens than in normal cartilage [8] but also increasingly higher levels of MMP-2 expression in human chondrosarcoma cells stimulate their migratory and metastatic potential [7,8]. Interestingly, microtubule depolymerizing drugs display antitumor activities in soft tissue sarcomas [9] while the transcription factor ETV5 reportedly regulates MMP-2 expression in human chondrosarcomas [10]. Chemokine (C-C motif) ligand 3 (CCL3) has been found to increase MMP-2 expression in human chondrosarcoma cells and thus encourage their migratory abilities while the inhibition of MMP-2 expression abolishes this effect of CCL3 [8]. Moreover, the anti-inflammatory drug zaltoprofen inhibits the proliferation, migration and invasion of chondrosarcoma cells by reducing the MMP-2 activity [11]. It therefore seems reasonable to speculate that inhibiting MMP-2 expression would be a useful therapeutic tactic in chondrosarcoma metastasis.

MicroRNAs (miRNAs) are involved in the cellular processes of different human diseases including cancer, cardiovascular disease and arthritic diseases [12,13,14,15] where they regulate different activities of the tumor cell including apoptosis, proliferation, angiogenesis, drug resistance and metastasis [16]. The importance of miRNAs in tumorigenesis is underlined by the fact that they perpetuate the process by targeting key metabolic enzymes and protein messenger RNAs (mRNAs) [17]. In particular, with regard to lung cancer cellular metabolism, researchers have suggested that miRNA mimics or inhibitors of metabolic processes and gene regulatory events could improve the overall survival in lung cancer [17]. Recently, miRNAs levels have been suggested to serve as potential biomarkers and therapeutic targets in cancer [18]. In addition, miRNAs are regulated in different roles of the tumor cell [16]. Interestingly, the evidence suggests that suppressing miR-101 and MMP-2 expression in human chondrosarcoma cells inhibits chondrosarcoma metastasis to the lungs [19].

Nerve growth factor (NGF) plays a critical role in neuronal cell growth, apoptosis and differentiation [20]. The binding of NGF to its receptor, tropomyosin receptor kinase A (TrkA), activates intracellular signaling and immune cell proliferation, differentiation and survival [21]. Several reports have suggested that NGF plays an integral part in the progression of several types of malignancies such as ovarian, prostate and liver cancers [22,23,24]. NGF also mediates metastasis in several types of tumors [24,25,26]. However, the role of NGF in a chondrosarcoma is unknown. In this study, we found that NGF promotes chondrosarcoma metastasis in vitro and in vivo. NGF also promotes MMP-2-dependent migration and invasion by inhibiting miR-423-5p expression through the FAK and c-Src signaling cascades.

## 2. Materials and Methods

### 2.1. Materials

NGF, MMP-2, FAK, c-Src and β-actin antibodies were obtained from GeneTex International Corporation (Hsinchu City, Taiwan). The phosphorylated forms of FAK (p-FAK) and c-Src (p-c-Src) antibodies were purchased from Cell Signaling Technology (Danvers, MA, USA). MMP-2, FAK, c-Src and control ON-TARGETplus siRNAs were obtained from Dharmacon (Lafayette, CO, USA). Quantitative polymerase chain reaction (qPCR) primers and probes, as well as Taqman^®^ One-Step PCR Master Mix, were supplied by Applied Biosystems (Foster City, CA, USA). Recombinant human NGF was obtained from PerpoTech (Rocky Hill, NJ, USA). An ABC Kit was obtained from Vector Laboratories (Burlingame, CA, USA). The human chondrosarcoma tissue array was purchased from Biomax (OS802c; Rockville, MD, USA) and the detailed clinical data are presented in Supplementary Table S1. Study approval was granted by the local Institutional Review Board (CMUH107-REC3-165). All other chemicals used in this study were supplied by Sigma-Aldrich (St. Louis, MO, USA).

### 2.2. Cell Culture

SW1353 chondrosarcoma cells were bought from ATCC (Manassas, VA, USA); JJ012 chondrosarcoma cells were obtained from Dr. Sean P. Scully (University of Miami; Miami, FL, USA). JJ012 cells stably expressing the NGF complementary DNA (cDNA) clone (JJ012/NGF cells) were established according to our previous method [27]. The cells were cultured 50%/50% in a Dulbecco’s Modified Eagle Medium (DMEM)/alpha-minimum essential medium (α-MEM) medium, 10% fetal bovine serum (FBS) and antibiotics then maintained in a humidified incubator at 37 °C in 5% CO2.

### 2.3. Cell Migration Assay

The chondrosarcoma cells were seeded into the upper chamber of Transwell plates (Costar; Corning, NY, USA) while NGF and pharmaceutical inhibitors were added to the lower chamber. After 18 h of incubation, the migrated cells were fixed with 3.7% formaldehyde and stained with crystal violet then counted manually under the microscope [28,29].

### 2.4. Wound Healing Assay

The confluent chondrosarcoma monolayer was scratched by a fine pipette tip to create extended scratches in each well. The cells were then treated with the conditions as indicated, migratory activity was evaluated by microscopy after 24 h and the rate of the wound closure was quantified [30].

### 2.5. Western Blot Analysis

After the indicated treatments, the chondrosarcoma cells were lysed in a RIPA buffer. The extracted proteins were resolved by SDS-PAGE and transferred to Immobilon^®^ polyvinylidene fluoride (PVDF) membranes. A Western blot analysis was performed using the methodology described in our previous reports [31,32,33].

### 2.6. mRNA and miRNA Quantification

Total RNA was extracted from the chondrosarcoma cells using a TRIzol reagent and RNA concentrations were determined using a NanoVue Plus spectrophotometer (GE Healthcare Life Sciences; Pittsburgh, PA, USA). The M-MLV RT kit (Thermo Fisher Scientific; Waltham, MA, USA) and the Mir-X™ miRNA First-Strand Synthesis kit (Clontech; Mountain View, CA, USA) were used to perform the reverse transcription of the total RNA into cDNA. A quantitative real-time PCR (qPCR) analysis was performed according to our previous reports [34,35].

### 2.7. Luciferase Assay

The human MMP-2 luciferase reporter plasmids containing wild-type or mutant sequences of the three prime untranslated region (3′-UTR) encompassing miR-423-5p binding sites were obtained from MDBio, Inc. (Taipei, Taiwan). The chondrosarcoma cells were transfected with the plasmids using Lipofectamine 2000 (Thermo Fisher Scientific; Waltham, MA, USA) then stimulated with NGF for 24 h. The luciferase activity was monitored using a luciferase assay kit [34,36,37].

### 2.8. Tumor Xenograft study

Four-week-old male BALB/c nude mice (eight in each group) were bought from Taipei’s National Laboratory Animal Center and orthotopically injected with JJ012 or JJ012/NGF cells (5 × 10^6^, resuspended in 100 μL of a medium containing 50% serum-free DMEM/α-MEM and 50% Matrigel) according to a previous protocol [27]. The tumor growth in the tibiae was monitored each week by bioluminescence imaging using a Xenogen IVIS imaging system 200 (PerkinElmer; Waltham, MA, USA). At 12 weeks, the mice were euthanized by CO2 inhalation. The lungs were removed and fixed in 10% formalin for a further analysis. All animal experiments satisfied the protocols specified by China Medical University’s Institutional Animal Care and Use Committee (IACUC Approval No. 104-154-N).

### 2.9. Immunohistochemistry (IHC) Staining

Mouse lung tissues or specimens from a human chondrosarcoma tissue array were rehydrated and incubated with primary anti-NGF or MMP-2 antibodies. The tissues were then incubated with a secondary antibody using the ABC kit. The IHC staining intensity was independently scored by two pathologists blinded to the study results as 0, 1+, 2+, 3+, 4+ or 5+ for the absence of staining, very weak, weak, moderate, strong or very strong staining, respectively.

### 2.10. Statistics

Data are presented as the mean ± standard deviation (SD). All differences between the groups were assessed for significance using the Student’s *t*-test. A *p* value of <0.05 was considered significant.

## 3. Results

### 3.1. NGF and MMP-2 Levels Are Positively Correlated in Human Chondrosarcoma Tissue

**Figure 1:**
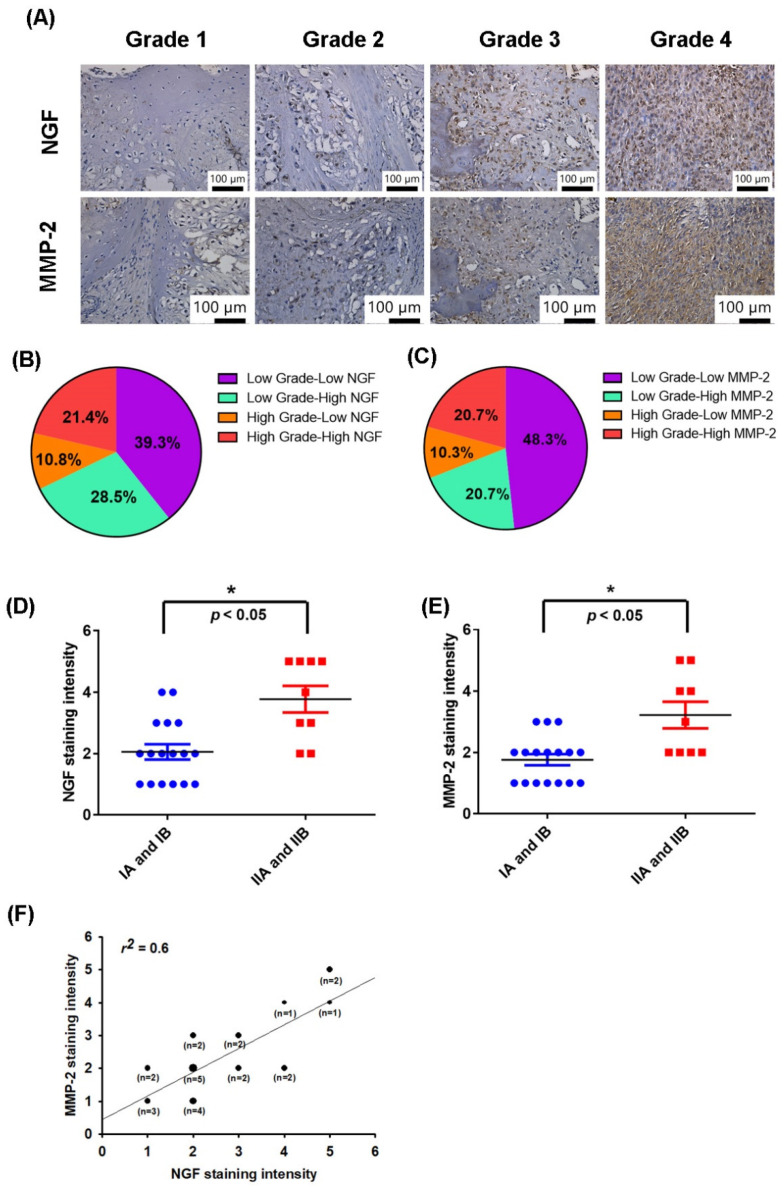
NGF and MMP-2 levels correlated with the chondrosarcoma tumor stage. (**A**–**E**) Immunohistochemistry (IHC)-stained tissue samples from chondrosarcoma patients were stained with NGF and MMP-2 antibodies then photographed and quantified. (**F**) Levels of NGF and MMP-2 were positively correlated. * *p* < 0.05 compared with the early stage (IA and IB) tumor group.

NGF is associated with progression and survival in several cancer types [38,39]. MMP-2 has been reported to control the migration and metastasis of chondrosarcomas [8,40]. The IHC tissue array results revealed higher levels of NGF and MMP-2 expression in patients with a higher grade chondrosarcoma than in those with a lower grade disease; the levels of NGF and MMP-2 expression were reflected by the tumor stage (Figure 1A–C). These results are quantified in Figure 1D,E, which illustrate how the levels of NGF and MMP-2 expression were significantly higher in the higher stage tumors (IIA and IIB) than in lower stage tumors (IA and IB). A positive correlation observed between the MMP-2 and NGF staining intensity of the human chondrosarcoma tissue (r^2^ = 0.6, Figure 1F) indicated that the levels of these proteins were associated with the progression of chondrosarcoma disease.

### 3.2. NGF Promotes MMP-2-Dependent Migration via the FAK and c-Src Pathways in Chondrosarcoma

**Figure 2:**
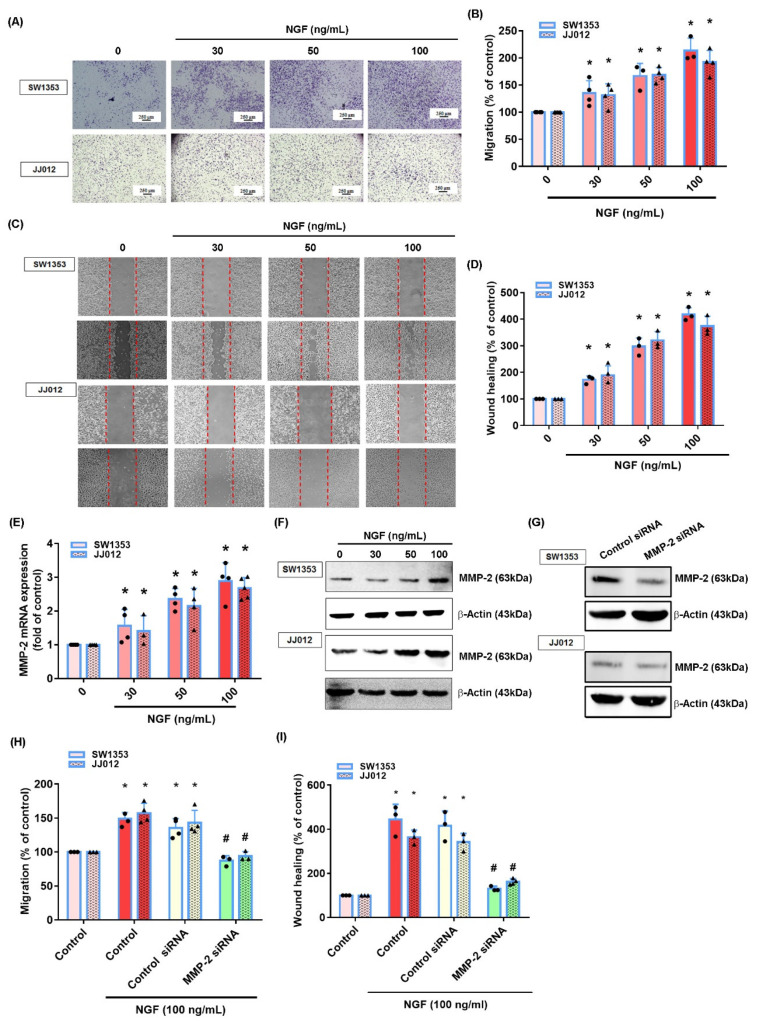
NGF promotes MMP-2-dependent cell migration in a human chondrosarcoma. (**A**–**D**) Cells were incubated with NGF (30–100 ng/mL) and the cell migration was examined by Transwell and wound healing assays. (**E**,**F**) Cells were incubated with NGF (30–100 ng/mL) and the levels of MMP-2 mRNA and protein expression were examined by qPCR and Western blot assays. (**G**–**I**) Cells were transfected with MMP-2 siRNAs then stimulated with NGF. The cell migration and MMP-2 expression levels were examined by Transwell, wound healing and Western blot assays. * *p* < 0.05 compared with the control group; # *p* < 0.05 compared with the NGF-treated group.

We first investigated the effects of NGF upon cell motility in chondrosarcoma cell lines SW1353 and JJ012. The treatment of cells with NGF promoted the migration ability, according to Transwell and wound healing assay data (Figure 2A–D). We then examined whether MMP-2 played a role in NGF-regulated migration in a chondrosarcoma. The stimulation of cells with NGF enhanced the mRNA and protein synthesis of MMP-2 (Figure 2E,F). The transfection of cells with MMP-2 siRNA diminished the NGF-induced promotion of migration (Figure 2G–I), implying that MMP-2 was critical to NGF-induced chondrosarcoma cell migration.

**Figure 3:**
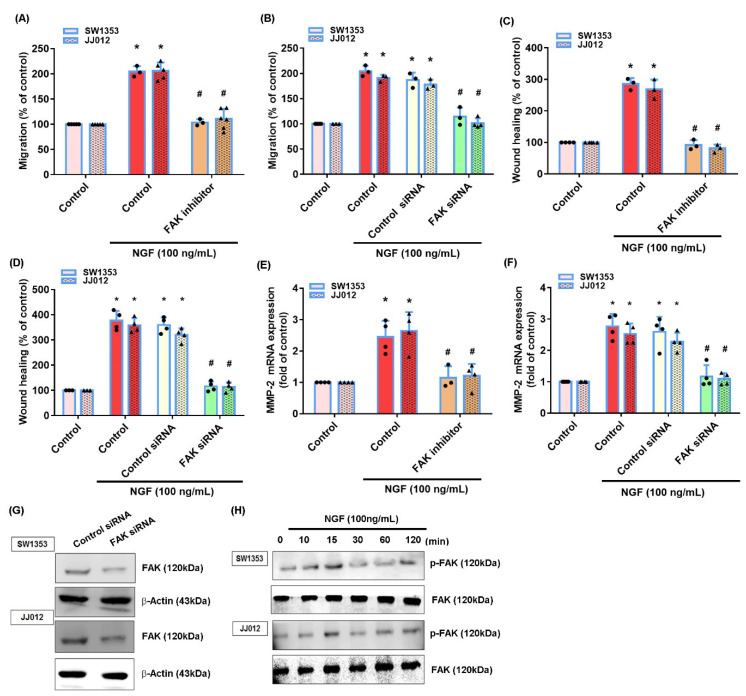
The FAK pathway mediates NGF-induced MMP-2 expression and cell migration. (**A**–**F**) Cells were pretreated with a FAK inhibitor or transfected with a FAK siRNA then stimulated with NGF. The cell migration and levels of MMP-2 expression were examined by Transwell, wound healing and qPCR. (**G**) Cells were transfected with a FAK siRNA and FAK expression was examined by Western blot. (**H**) Cells were incubated with NGF for the indicated time intervals; FAK phosphorylation was examined by Western blot. * *p* < 0.05 compared with the control group; # *p* < 0.05 compared with the NGF-treated group.

**Figure 4:**
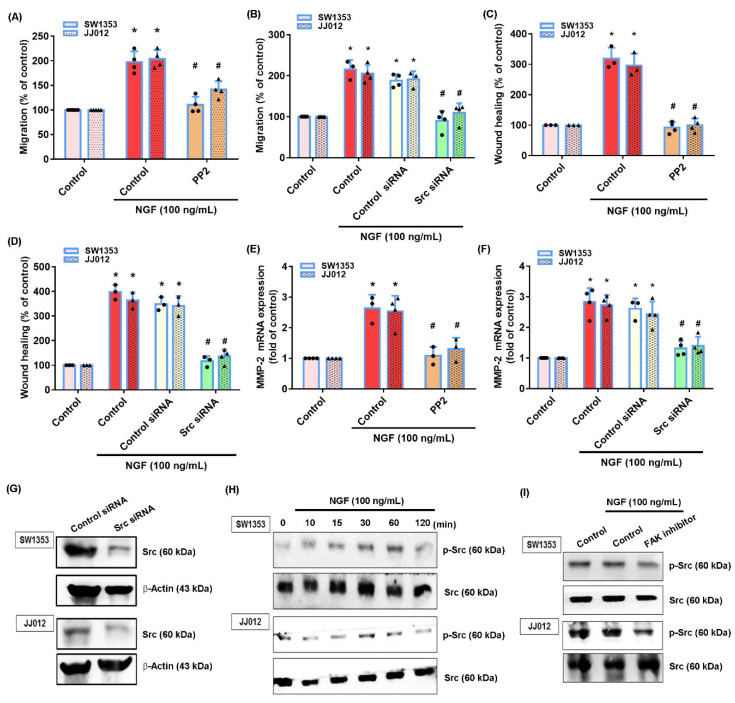
The c-Src pathway mediates NGF-induced MMP-2 expression and cell migration. (**A**–**F**) Cells were pretreated with a c-Src inhibitor (PP2) or transfected with a c-Src siRNA then stimulated with NGF. The cell migration and levels of MMP-2 expression were examined by Transwell, wound healing and qPCR assays. (**G**) Cells were transfected with a c-Src siRNA and c-Src expression was examined by Western blot. (**H**,**I**) Cells were incubated with NGF for the indicated time intervals or pretreated with a FAK inhibitor then stimulated with NGF; c-Src phosphorylation was examined by Western blot. * *p* < 0.05 compared with the control group; # *p* < 0.05 compared with the NGF-treated group.

The FAK and c-Src signaling pathway plays a critical role in chondrosarcoma metastasis [41]. Treating cells with a FAK inhibitor or c-Src inhibitor (PP2) significantly reduced the NGF-induced stimulation of cell migration and MMP-2 production (Figure 3A–F and Figure 4A–F). Similar effects were observed when the chondrosarcoma cells were transfected with FAK or c-Src siRNAs (Figure 3A–G and Figure 4A–F). NGF stimulation time-dependently promoted the FAK and c-Src phosphorylation in both cell lines (Figure 3H and Figure 4H). Treating cells with a FAK inhibitor diminished the NGF-induced c-Src phosphorylation (Figure 4I) indicating that the FAK/c-Src signaling cascade regulated the NGF-induced MMP-2 synthesis and chondrosarcoma cell migration.

### 3.3. The miR-423-5p/MMP-2 Axis Controls the NGF-Induced Stimulation of Chondrosarcoma Cell Migration

**Figure 5:**
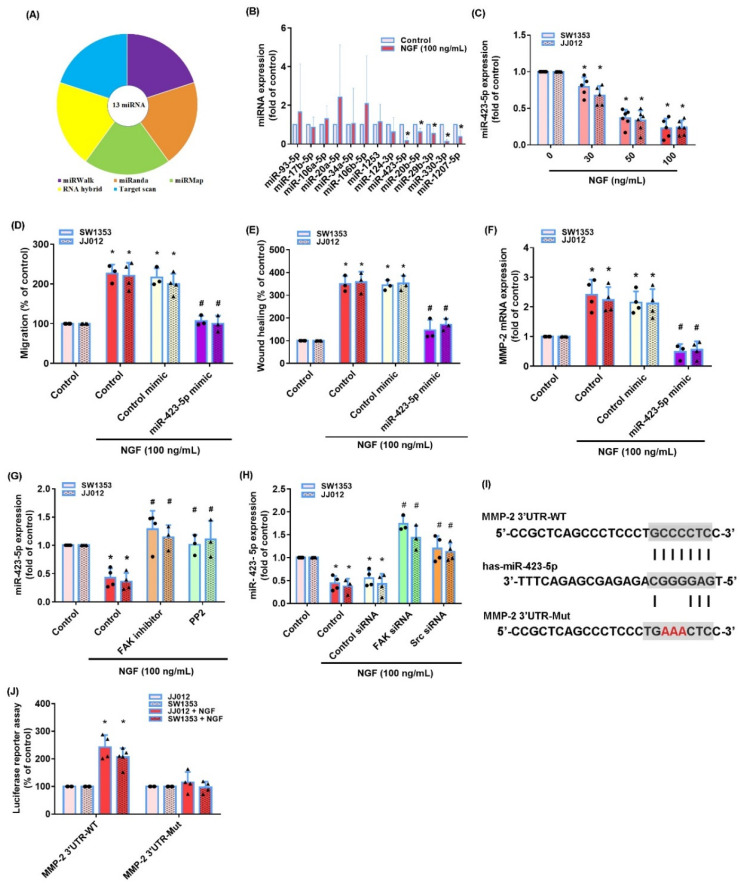
Inhibition of miR-423-5p mediates the NGF-induced stimulation of MMP-2 expression and the migratory activity of human chondrosarcoma cells. (**A**,**B**) MiRNA target prediction software was used to identify miRNAs that potentially bind to the MMP-2 3′-UTR. (**C**) SW1353 and JJ012 cells were incubated with NGF and miR-423-5p levels were examined by qPCR. (**D**–**F**) Cells were transfected with an miR-423-5p mimic then stimulated with NGF. The cell migration and MMP-2 expression levels were examined by Transwell, wound healing and qPCR. (**G**,**H**) Cells were pretreated with FAK and c-Src inhibitors or an siRNA and then stimulated with NGF prior to a qPCR analysis of the miR-423-5p levels. (**I**) The wild-type and mutant MMP-2 3′-UTRs contained the miR-423-5p binding site. (**J**) Cells were transfected with 3′-UTR plasmids as indicated then stimulated with NGF. The luciferase activity was examined. * *p* < 0.05 compared with the control group; # *p* < 0.05 compared with the NGF-treated group.

The miRNA-associated regulation of MMP-2 expression is a critical mechanism in the development, progression, migration and metastasis of cancer cells [42]. Five online databases for miRNA target predictions indicated that the 3′-UTR region of MMP-2 mRNA contains 13 promising candidate miRNAs (Figure 5A,B). The treatment of JJ012 cells with NGF (100 ng/mL) significantly reduced miR-423-5p expression (Figure 5B) and at the concentrations of 30, 50 or 100 ng/mL, significantly inhibited the miR-423-5p synthesis in both chondrosarcoma cell lines in a concentration-dependent manner (Figure 5C). The transfection of cells with an miR-423-5p mimic significantly reduced the NGF-induced stimulation of the cell migration and MMP-2 mRNA expression in both chondrosarcoma cell lines (Figure 5D–F). We then investigated whether FAK and c-Src signaling regulated the NGF-induced suppression of the miR-423-5p synthesis. The FAK and c-Src inhibitors and their respective siRNAs all reversed the NGF-induced inhibition of miR-423-5p expression (Figure 5G,H). Analyses of the MMP-2 3′-UTR luciferase plasmids revealed that NGF increased the luciferase activity of the wild-type but not the mutant MMP-2 3′-UTRs (Figure 5I,J). These results indicated that miR-16-5p controlled MMP-2 expression by anchoring to the 3′-UTR region of the human MMP-2 gene via the FAK/c-Src pathway.

### 3.4. The Overexpression of NGF Facilitates Chondrosarcoma Metastasis of the Lungs in Mice

**Figure 6:**
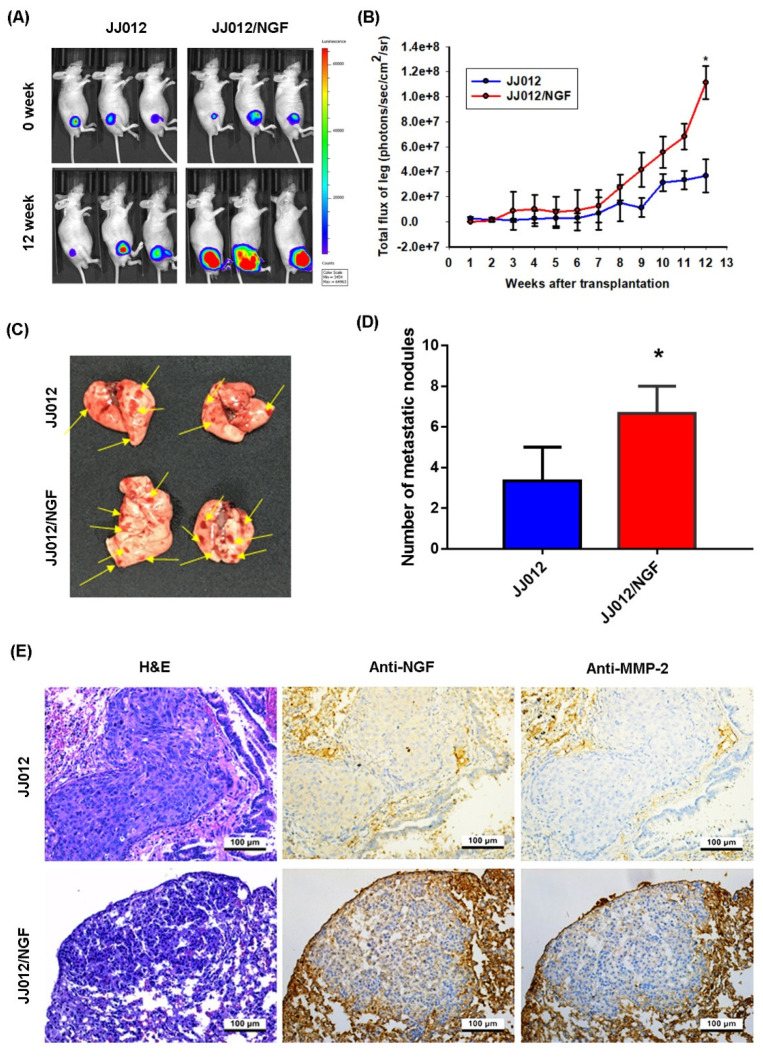
NGF promotes chondrosarcoma metastasis to the lungs in vivo. (**A**,**B**) The mice were injected with JJ012 or JJ012/NGF cells. The lung metastasis was monitored by bioluminescence imaging at the indicated time intervals then quantified by photon images. (**C**,**D**) After 12 weeks, the mice were humanely sacrificed and the lung tissue was excised, photographed and quantified. (**E**) The levels of NGF and MMP-2 expression in the lung tumors were subjected to an IHC analysis. * *p* < 0.05 compared with the control group.

We used the orthotopic in vivo model of chondrosarcoma lung metastasis to further investigate the promoting effects of NGF in a metastatic chondrosarcoma [27]. JJ012 and JJ012/NGF cells were orthotopically implanted into the right leg tibia and the tumor size was monitored each week by the IVIS system (Figure 6A,B). The overexpression of NGF significantly increased the tumor growth in the tibia (Figure 6A,B). At 12 weeks, metastasis to the lung was significantly more likely with JJ012/NGF cells than with JJ012 cells (Figure 6C,D). The IHC results revealed significant increases in the levels of NGF and MMP-2 expression in the JJ012/NGF orthotopic model (Figure 6E), confirming that NGF facilitated the metastasis of the chondrosarcoma to the mouse lung.

## 4. Discussion

A chondrosarcoma is a malignant bone neoplasm that constitutes almost one-third (~26%) of all bone cancers [43]. Chemotherapy and radiotherapy have very limited effectiveness so treatment with surgery is therefore the major management modality for a chondrosarcoma. This malignancy is notorious for its aggressive clinical course and propensity to metastasize [44]. An effective adjuvant therapy is urgently needed to suppress the metastasis of chondrosarcomas [1,3]. NGF plays an important role in tumor cell proliferation, migration and survival [38,39]. The effect of NGF in a chondrosarcoma metastasis is uncertain. Our investigation has found that levels of NGF and MMP-2 expression are positively correlated with the tumor staging in patients with a chondrosarcoma. We also confirmed that NGF facilitates MMP-2-dependent chondrosarcoma cell migration and metastasis by inhibiting the miR-423-5p synthesis via FAK/c-Src signaling.

NGF acts not only on the central and peripheral nervous systems but also on non-neuronal tissues and cancer cells [38]. NGF plays a multi-functional role in the tumor environment, exerting effects on tumor cell proliferation, survival, apoptosis, angiogenesis and metastasis [38,45,46]. Our study is the first to describe an association between NGF levels and the tumor stage in chondrosarcoma tissue specimens. The evidence from the in vitro and in vivo results suggested that NGF facilitates the chondrosarcoma metastasis. The binding of NGF with the neurotrophin receptor TrkA mediates the NGF control in the development of cancer [47]. The knockdown of the TrkA receptor suppresses the progression of liver cancer [48] and a Trk receptor inhibitor antagonizes NGF-induced cell motility in prostate cancer [49]. These results suggest that the inhibition of the TrkA receptor reduces NGF-mediated cancer development. In this study, we did not examine the TrkA receptor levels in patients with a chondrosarcoma. Whether TrkA inhibition antagonizes the NGF-mediated chondrosarcoma metastasis is yet to be confirmed.

The activation of the FAK/c-Src pathway is important in the regulation of different cellular functions [50,51]. This signaling pathway also regulates the expression of MMP-mediated cancer motility [52,53]. Here, our results showed that NGF promoted the phosphorylation of FAK and c-Src while FAK and c-Src pharmacological inhibitors suppressed the NGF-induced promotion of MMP-2 expression and the chondrosarcoma migration. This phenomenon was confirmed by similar effects observed with genetic siRNAs of FAK and c-Src. However, c-Src has been reported to be an upstream molecule of FAK with the capacity to regulate cell motility [54,55]. Here, we found that a FAK inhibitor curtailed the NGF-promoted phosphorylation of c-Src, indicating that FAK activated c-Src.

MiRNAs post-transcriptionally regulate gene expression [56]. During a tumor metastasis, aberrant miRNA expression mediates the cancer cell migration and invasion [57]. Our analysis of five open-source databases identified that 13 miRNAs potentially interfere with MMP-2 transcription. NGF significantly lowered miR-423-5p expression. We enhanced miR-423-5p levels in chondrosarcoma cells by transfecting them with a specific miR-423-5p mimic, which also reduced the MMP-2 synthesis and the migratory capacity of the cells. miR-423-5p expression was negatively correlated with MMP-2 expression and the cell migratory activity in the chondrosarcoma cells. Thus, our evidence identified novel antimetastatic properties of miR-423-5p.

With regard to the limitations of this study, the human chondrosarcoma tissue array contained samples from 26 patients, 9 of whom had higher stage (IIA and IIB) tumors and 17 had lower stage (IA and IB) tumors, which did not provide a sufficiently large enough sample to avoid false positive conclusions. Moreover, as these tissue arrays do not provide information about neoadjuvant treatment, chemotherapy and radiotherapy, we could not perform a detailed analysis on NGF and MMP-2 levels in relation to the tumor stage. We recommend that future studies enroll larger clinical samples.

## 5. Conclusions

**Figure 7:**
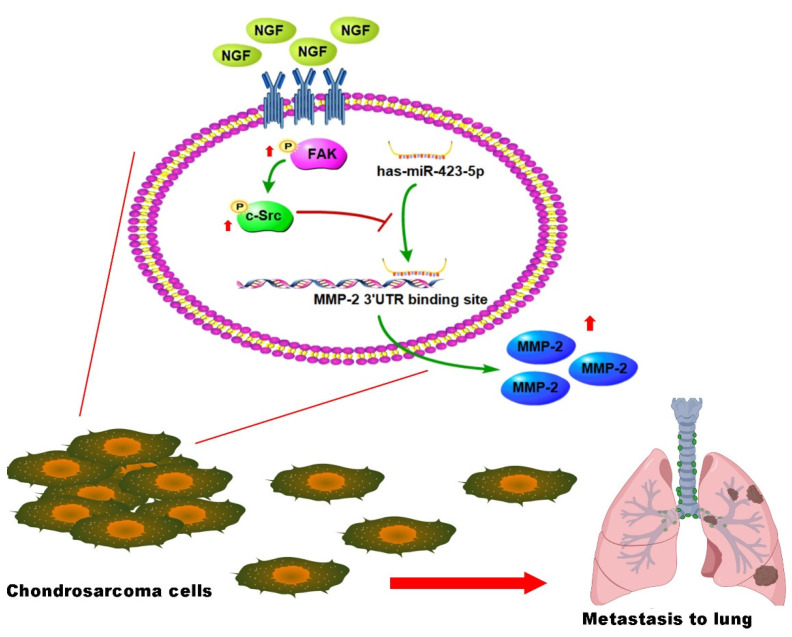
Schema illustrating the effects of NGF in a chondrosarcoma metastasis. NGF facilitates the MMP-2-dependent migratory activities of the chondrosarcoma cells and metastasis by inhibiting the miR-423-5p synthesis via the FAK and c-Src signaling cascades.

In conclusion, our study identified that NGF promoted the MMP-2-dependent cell migration in human chondrosarcoma tissue by inhibiting the miR-423-5p synthesis via the FAK and c-Src signaling cascades (Figure 7). It appears to be worth targeting NGF expression in a metastatic chondrosarcoma.

## Data Availability

The datasets used and/or analyzed during this study are available from the corresponding authors upon reasonable request.

## Supplementary Materials

The following are available online at https://www.mdpi.com/article/10.3390/cancers13133347/s1, Table S1: Chondrosarcoma tissue array detailed information.
