# Supplementary material for: The mir-423-5p/MMP-2 Axis Regulates the Nerve Growth Factor-Induced Promotion of Chondrosarcoma Metastasis
Source: Cancers (Basel). 2021 Jul 3;13(13):3347. doi: 10.3390/cancers13133347 (PMC8268073; doi:10.3390/cancers13133347)
Supplement: Supplementary file 1 [file cancers-13-03347-s001.zip › cancers-1263136-supplementary.pdf]

Supplementary Materials

The mir-423-5p/MMP-2 Axis Regulates the Nerve Growth Factor-Induced Promotion of Chondrosarcoma Metastasis

Huey-En Tzeng, Syuan-Ling Lin, Louis Anoop Thadevoos, Chih-Yuan Ko, Ju-Fang Liu, Yu-Wen Huang, Chih-Yang Lin, Yi-Chin Fong and Chih-Hsin Tang

Table S1. Chondrosarcoma tissue array detailed information from the US Biomax, Inc. (OS802c), including TNM and clinical stage.

No.	Age	Sex	Organ/ Anatomic Site	Pathology diagnosis	TNM	Grade	Stage	Type
**1**	22	M	Cartilage	Well differentiated chondrosarcoma of left thigh	T1N0M0	G1	IA	Malignant
**2**	37	M	Cartilage	Well differentiated chondrosarcoma of left ilium	T1N0M0	G1	IA	Malignant
**3**	39	F	Cartilage	Well differentiated chondrosarcoma of right scapula	T1N0M0	G1	IA	Malignant
**4**	40	M	Cartilage	Well differentiated chondrosarcoma	T1N0M0	G1	IA	Malignant
**5**	47	F	Cartilage	Well differentiated chondrosarcoma of left shoulder	T1N0M0	G1	IA	Malignant
**6**	50	M	Cartilage	Well differentiated chondrosarcoma of left thigh	T1N0M0	G1	IA	Malignant
**7**	50	M	Cartilage	Well differentiated chondrosarcoma of L3 vertebrae	T1N0M0	G1	IA	Malignant
**8**	50	M	Cartilage	Well differentiated chondrosarcoma of femur	T1N0M0	G1	IA	Malignant
**9**	52	M	Cartilage	Well differentiated chondrosarcoma of sacroiliac	T1N0M0	G1	IA	Malignant
**10**	64	F	Cartilage	Well differentiated chondrosarcoma of back	T1N0M0	G1	IA	Malignant
**11**	74	M	Cartilage	Well differentiated chondrosarcoma of right middle finger	T1N0M0	G1	IA	Malignant
**12**	14	M	Bone	Moderately differentiated chondrosarcoma of right tibia	T2N0M0	G2	IB	Malignant
**13**	16	M	Bone	Chondrosarcoma of tibia	T2N0M0	G2	IB	Malignant
**14**	27	F	Bone	Well differentiated chondrosarcoma of left femur	T2N0M0	G1	IB	Malignant
**15**	35	M	Cartilage	Well differentiated chondrosarcoma of left ilium	T2N0M0	G1	IB	Malignant
**16**	42	M	Cartilage	Moderately differentiated chondrosarcoma of ankle	T2N0M0	G2	IB	Malignant
**17**	59	M	Cartilage	Well differentiated chondrosarcoma of right thumb	T2N0M0	G1	IB	Malignant
**18**	27	M	Bone	Poorly differentiated chondrosarcoma of left tibia	T1N0M0	G3	IIA	Malignant
**19**	28	M	Cartilage	Dedifferentiation of chondrosarcoma of left sole of foot	T1N0M0	G4	IIA	Malignant
**20**	30	M	Cartilage	Mesenchymal chondrosarcoma of leg	T1N0M0	G4	IIA	Malignant
**21**	35	M	Cartilage	Poorly differentiated chondrosarcoma of left femur	T1N0M0	G3	IIA	Malignant
**22**	38	M	Cartilage	Dedifferentiation of chondrosarcoma of left hip	T1N0M0	G4	IIA	Malignant
**23**	41	M	Cartilage	Chondrosarcoma of femur	T1N0M0	G3	IIA	Malignant
**24**	48	F	Cartilage	Chondrosarcoma of femur	T1N0M0	G3	IIA	Malignant
**25**	12	F	Cartilage	Chondrosarcoma of right femur	T2N0M0	G3	IIB	Malignant
**26**	13	F	Bone	Poorly differentiated chondrosarcoma of left tibia	T2N0M0	G3	IIB	Malignant

TNM grading: T1—Tumor invades submucosa, T2—Tumor invades muscularis propria.
